# Effect of high temperature on *Wolbachia* density and impact on cytoplasmic incompatibility in confused flour beetle, *Tribolium confusum* (Coleoptera: Tenebrionidae)

**DOI:** 10.1186/s13104-022-06123-y

**Published:** 2022-07-07

**Authors:** Yeganeh Gharabigloozare, Christoph Bleidorn

**Affiliations:** Department of Animal Evolution and Biodiversity, Johann-Friedrich-Blumenbach Institute for Zoology and Anthropology, University of Göttingen, Göttingen, Germany

**Keywords:** *Wolbachia* density, Fertility, Cytoplasmic incompatibility, Heat stress

## Abstract

**Objectives:**

Environmental constraints, especially temperature, have been identified as a key in understanding host-symbiont relationships, as they can directly impact the fitness of the symbiont population and the host development. Here we investigated the effect of temperature during the host development on the density of intracellular bacteria of the *Wolbachia*, *w*Tcon strain within the confused flour beetle, *Tribolium confusum*. The *w*Tcon can induce a complete cytoplasmic incompatibility (CI) in *T. confusum* beetles; therefore, we observed the effect of heat stress on the symbiont-mediated CI.

**Results:**

The density of CI inducing *Wolbachia* in the *Tribolium confusum* is temperature-specific. Our observation of the beetles reared in five different temperatures (30–34 °C) measured the highest *Wolbachia* density at 30–31 °C and lowest at 34 °C within a single insect generation. In this species, changes in the density of *Wolbachia* related to higher temperature did not influence CI. However, the fertility of beetles reared in higher temperatures showed a substantial decrease in the number of laid and hatched eggs. Thus, we can confirm the effect of high temperature on lowering the *w*Tcon density and no impact on induction of cytoplasmic incompatibility (CI) in *T. confusum* beetles.

**Supplementary Information:**

The online version contains supplementary material available at 10.1186/s13104-022-06123-y.

## Introduction

Environmental factors are of primary importance in the development and survival of the host-endosymbiont relationship. In particular, the temperature directly impacts the ecological and evolutionary dynamics of populations and the individual’s infection development and pathogen virulence [1, 2]. The influence of temperature in various symbiotic model systems may help predict the evolution of local adaptations to regulate infection density [3]. However, the temperature can have a specific effect on both the symbiont and the host separately; the influence on the symbiotic interaction relies on the type of symbiosis between host and symbiont [1, 2]. For example, short exposure to high temperatures in pea aphids eliminates all or most of their bacterial symbiont, *Buchnera aphidicola*. This resulted in drastically lower fecundity and reduced thermal resistance of hosts due to a deficiency in the production of essential amino acids derived from the obligatory symbiont [4].

*Wolbachia*, arguably the most common animal endosymbiont in nature, is a maternally inherited intracellular bacteria belonging to Alphaproteobacteria, present in arthropods and nematodes [5–7]. *Wolbachia* lineages are classified into 17 supergroups (A–H) based on their divergence in molecular phylogenetic analyses, which differ in their host range and type of symbiosis, spanning from mutualistic to parasitic [8, 9]. The *Wolbachia*-host symbiosis can affect the host fitness, especially by manipulating reproduction, e.g., due to feminization, parthenogenesis, male-killing, or cytoplasmic incompatibility (CI) to eventually increase their spread in the host population [6, 10]. The effect of temperature on *Wolbachia* has always been of considerable interest, as it may influence endosymbiont density and completeness of CI [11], yet this effect may vary among endosymbiont strains and hosts. Previous studies showed that extremely high and low temperatures could be lethal for the symbiont [12, 13]. In *Drosophila bifasciata*, lower *Wolbachia* density has been recorded at elevated temperature (26 °C) [14]*;* however*, D. simulans* males favor low temperature (19 °C) in terms of the infection density, especially during larval development [15]. Interestingly, *Wolbachia* is even able to manipulate the temperature preference of its hosts [16].

Here we explore the effects of high temperature by comparing *Wolbachia* density in naturally infected (MN61) and uninfected (HP70) stocks of confused flour beetle, *Tribolium confusum*, from Kansas, USA. Both *T. confusum* stocks may differ in their genetic background, and as such, this could influence the fecundity of crosses between them. Previous studies demonstrated the presence of complete CI and reproductive isolation between the beetle populations [17]. One follow-up study reported the probability of the existence of two different *Wolbachia* strains [5]; nevertheless, Fialho and Stevens [18] showed that out of eight different stocks of *T. confusum*, all were infected with a single and common CI inducing strain [18–21]. Here, we investigated the effect of high temperature on the density of *Wolbachia* infection in *T. confusum* adults. High temperature has a significant impact on *Wolbachia* density, which might influence the host reproduction, and as such, we investigated (i) how symbiont density is affected by heat, (ii) the impact of heat on CI, and (iii) if high temperature influences sex ratio of the host regarding *Wolbachia* infection.

## Main text

### Methods

#### Insect biology and rearing

In this study, two stocks of *Tribolium confusum* (Coleoptera: Tenebrionidae) beetles were compared, being either infected (MN61) or uninfected (HP70) with *Wolbachia*. The beetle’s stock was established from adults and transported from the Stored Product Insect and Engineering Research center of USDA in Kansas, USA. They were stored in container boxes with a feed medium containing a small proportion of brewer yeast (5%) in type 405 wheat flour and maintained at 30 °C and 65 ± 5%RH under a 16:8 (D: L) cycle. Later, beetles were sexed at the pupal stage based on their urogomphi morphology [22].

#### DNA extraction and detection of *Wolbachia*

Single adults were removed from their stock containers for DNA extraction with Roboklon tissue and bacterial DNA kit (Roboklon GmbH, Berlin, Germany). Polymerase chain reaction (PCR) was carried out using primer pairs *wsp*F (5′-GCAGCATATATCAGCAATCCTTCAA) and *wsp*R (5′-GCATCATCCTTAGCCGCCTTAT) [20]. PCR was performed in thermocyclers in a total volume of 25 µl (12.5 µl DreamTaq PCR master mix, 0.5 µl for each forward and reverse primer, and 10.5 µl distilled water). The PCR thermal profile used was—one cycle (the 30 s 94 °C) followed by 35 cycles (15 s 94 °C, 30 s 53 °C) and one cycle (30 s 72 °C). Gel electrophoresis demonstrated DNA bands in 1% agarose gel stained in GelRed [23].

#### Effect of heat on *Wolbachia* density

Six young infected adults (3–5 days old—3 ♀ and 3 ♂), which were kept at the rearing temperatures of 30–34 °C, were tested to measure the relative *Wolbachia* density for two consecutive generations by carrying out real-time PCR on Rotor-Gene Q (Qiagen, Hilden, Germany). Each sample was pipetted two times into a 72-well plate and run with two sets of primers and two technical replicates for each sample. As for the first set, a specific pair for *Tribolium confusum* is *Tco*261F23 (CAGGATGAACTGTTTACC) and *Tco*474R25 (GTAGGTCGTATATTAATTACTG), along with a specific TaqMan probe (FAM-ATCATCTAATATCGCTCACGGAGGAG-TAMRA) to identify *T. confusum* were used. PCR amplification in a final reaction volume of 20 μl contained 10 μl Premix ExTaq (Probe qPCR, 2×) (ThermoFisher Scientific, MA, USA), 0.4 μl specific forward primer, 0.4 μl specific reverse primer, 0.8 μl TaqMan probe, 7.4 μl ddH2O, and 1 μl template DNA. The PCR cycler conditions were an initial denaturation at 95 °C for the 30 s, followed by 35 cycles of 95 °C for 5 s, 60 °C for 34 s, and a final extension at 72 °C for 10 min [24]. Other sets of primers were designed to detect *Wolbachia wsp* gene as *wsp*F (5′-GCAGCATATATCAGCAATCCTTCAA) and *wsp*R (5′-GCATCATCCTTAGCCGCCTTAT) as well as specific designed TaqMan probe (5′-FAM-TGTTAGCTATGATGTAAC135TCCAGAA-TAMRA). Real-time quantitative PCR reactions with a total volume of 20 μl contained 10 μl Premix ExTaq (Probe qPCR, 2×) (ThermoFisher Scientific, MA, USA), 0.2 μl specific forward primer, 0.2 μl specific reverse primer, 0.4 μl TaqMan probe, 8.4 μl ddH2O, and 2 μl template DNA. The temperature regime was as follows: 30 s at 95 °C for initial denaturation, then 40 cycles of 95 °C for 5 s, 60 °C for 34 s, and with a final extension at 72 °C for 10 min [21]. PCR was carried out to attain the crossing point (Cp) values for these markers of each beetle. Differences between the crossing point (ΔCp) of the *Wolbachia* and *Tribolium confusum* primers were calculated and then transformed by 2^n^ to reach the relative estimates of *Wolbachia* density [25].

#### Test for cytoplasmic incompatibility (CI)

Beetles reared at 30–34 °C were used to determine the effect of *Wolbachia* density on CI expression in incompatible crosses under heat stress. Crosses were performed in four combinations, using 3–5 days old males and virgin females (w^+^ × w^+^, w^+^ × w^−^, w^−^ × w^+^, and w^−^ × w^−^) with three cross-replicates per combination. After 3 days, the number of eggs for each cross and assigned temperature were calculated for 30 days. Subsequently, eggs were placed in separate vials containing flour medium and were checked for hatchability. The presence of CI and the level of incompatibility were estimated from this data using temperature as the only independent variable.

#### Test for reproduction and sex ratio

The results from the last experiment, and the numbers of emerging male and female adults (in the pupal stage), were recorded for each vial and temperature every day for 30 days. This data allowed us to estimate the reproduction and survival rate plus the sex ratio (% females) of beetles in five different temperatures.

#### Statistical analysis

For statistical analysis, one-way ANOVA and Tukey post-hoc tests were conducted in JMP v16.2.0 (SAS Institute Inc., Cary, NC, USA) to assess the effect of temperature on the density of endosymbiont bacteria, *Wolbachia* (Additional file 1), and also its impact on the fertility of *T. confusum* females. As for the sex ratio, the number of eggs for a pair of beetles was assessed for each temperature (mean ± SD) by the Tukey HSD test, p < 0.05 (Additional file 2).

### Results and discussion

#### Effect of heat on *Wolbachia* density

**Fig. 1:**
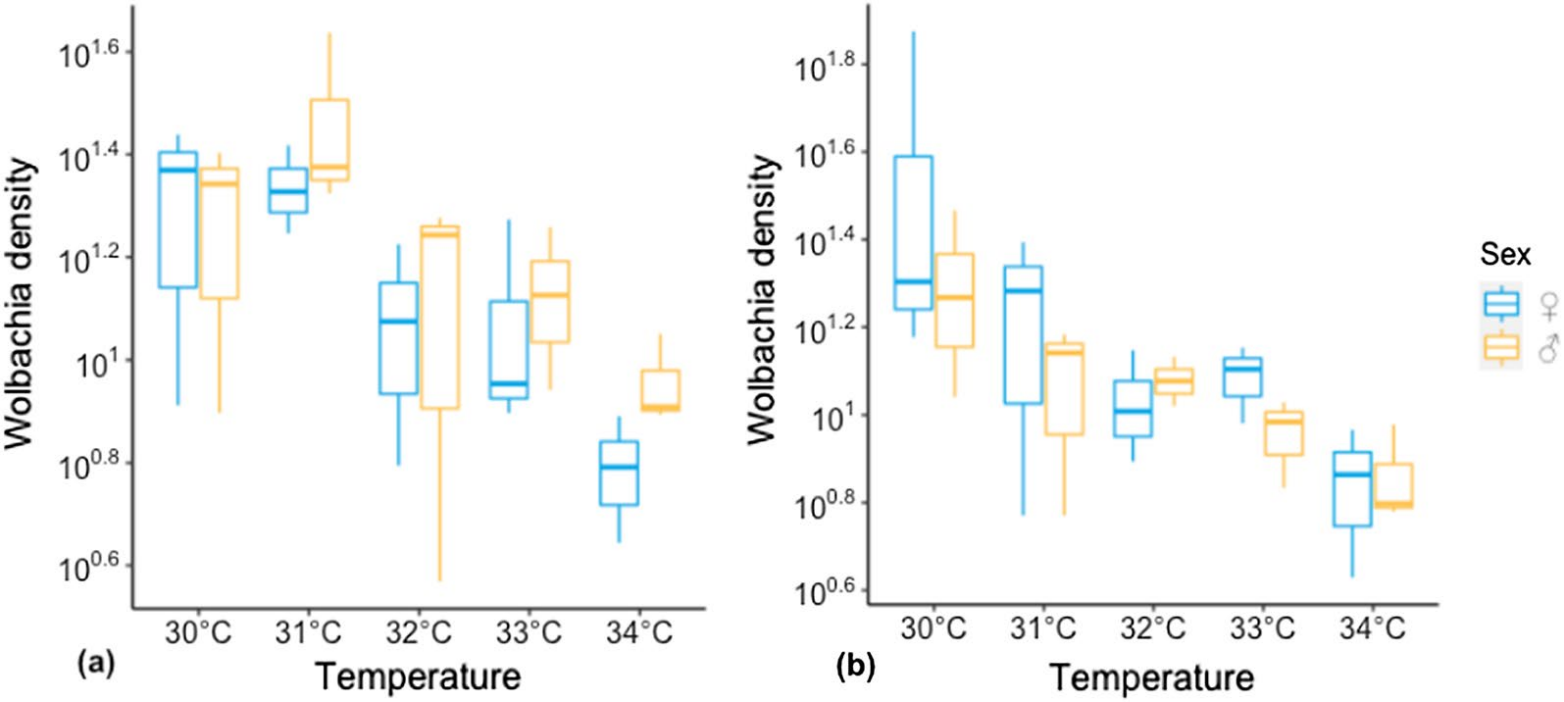
Relative density of *w*Tcon in *Tribolium confusum* reared under temperature cycles of 30–34 °C for two consecutive generations as F1 (**a**) and F2 (**b**)

Females and males of *T. confusum* were reared in five different temperatures from 30 °C as a favorable developmental temperature for the host to 34 °C, the highest temporal degree that the host survived. The relative density of *Wolbachia* in individual beetles varies with temperature. In two consecutive generations, the density of *Wolbachia* was not significantly different (F1: F_4,29_ = 5.61, p-value = 0.002, F2: F_9,29_ = 2.30, p-value = 0.057), however there was an obvious reduction of density for the beetles reared at 34 °C, in comparison with those reared at 30 °C (F1: p-value = 0.0352, F2: p-value = 0.001) and 31 °C (F1: p-value = 0.001) (Fig. 1).

Lu et al. [26] reported *Wolbachia* density differences between sexes, and we thus analyzed the results of the two sexes separately. However, our results showed no significant difference in the density of males regardless of the temperature that they reared at for two consecutive generations (F1: p-value = 0.46, F2: p-value = 0.45).

#### Impact of heat on cytoplasmic incompatibility

**Fig. 2:**
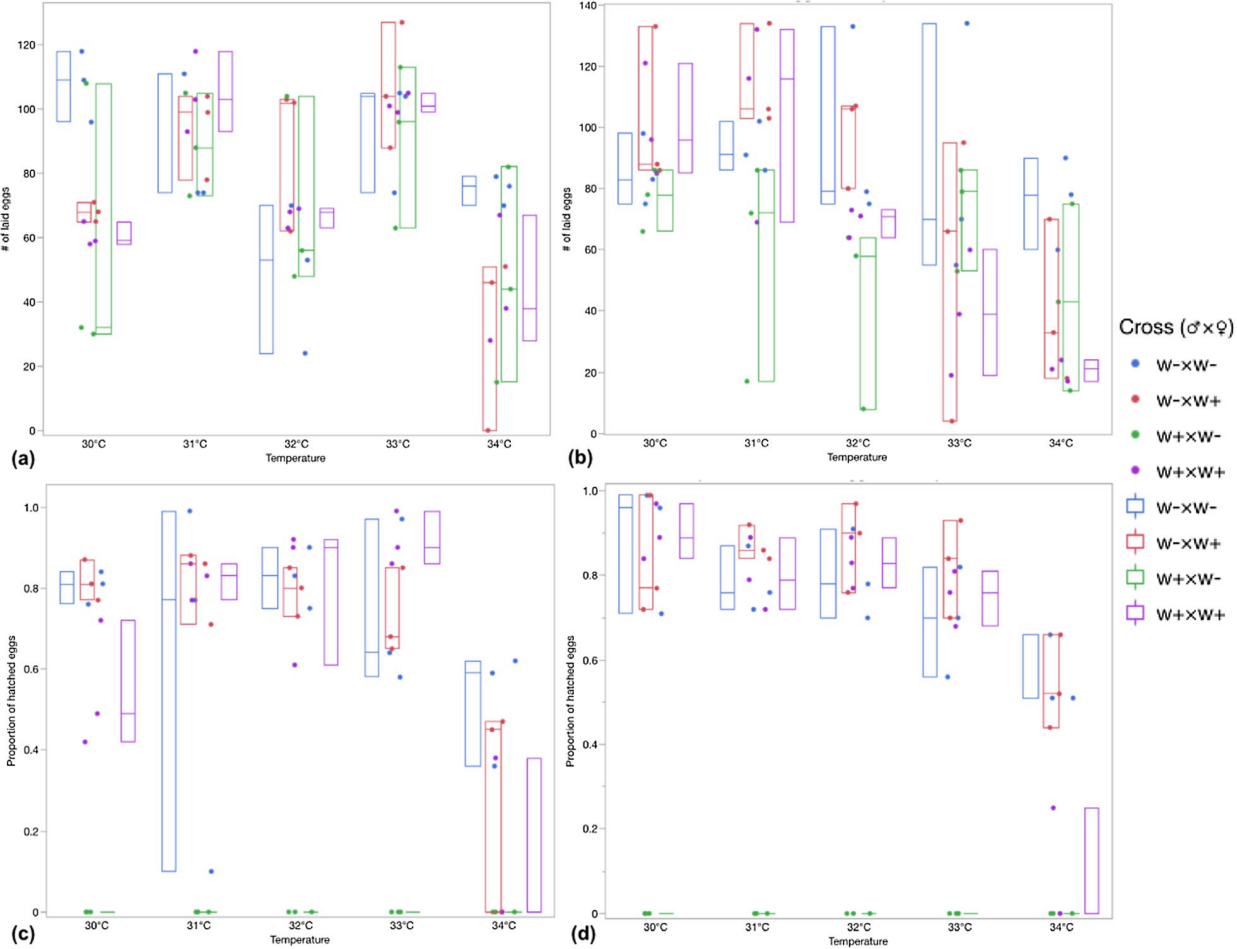
Box plots showing the effect of high temperature on the number of laid eggs for F1 (**a**) and F2 (**b**) in four cross combinations and hatch proportion in three cross combinations for F1 (**c**) and F2 (*d*) of *Tribolium confusum* beetles

Fecundity can be influenced by the temperature, so we first tested the effect of higher temperature on the fecundity of the virgin females and males in four different crosses respectively (w^+^ × w^+^, w^+^ × w^−^, w^−^ × w^+^, and w^−^ × w^−^). After counting the number of laid eggs for each cross in five different temperatures, no noticeable differences in the number of laid eggs in all four crosses were found (F1: F_4,59_ = 8.78, p-value < 0.001, F2: F_4,59_ = 5.795, p-value = 0.0006), except for an obvious decrease of egg production for the beetles reared at 34 °C, in comparison with those reared at 30 °C (F2: p-value = 0.001), 31 °C (F1: p-value = 0.026, F2: p-value = 0.001) and 33 °C (F1: p-value = 0.001) (Fig. 2a, b). However, temperature had no significant impact on the number of laid eggs in crosses of the beetles which were reared at 30–33 °C (F1: F_3,15_ = 2.256, p-value < 0.05, F2: F_3,15_ = 3.027, p-value < 0.05). In addition, the number of produced eggs for four different crosses regardless of what temperature they reared at, were not significant in both generations (F1: F_3,19_ = 0.53, p-value < 0.05, F2: F_3,19_ = 0.874, p-value < 0.05).

Afterward, we tested the effect of temperature on the completeness of CI. As for the number of the hatched eggs, the same results applied to the number of hatched eggs by a drastic reduction when reared at the highest survival temperature (34 °C) in all crosses (F1: F_4,44_ = 9.88, p-value < 0.0001, F2: F_4,44_ = 16.56, p-value < 0.0001). Although a study reported a reduced number of progenies and hatched eggs from the crosses between uninfected males and females in comparison with the other two crosses [21], our results cannot validate this finding (F1: F_2,44_ = 0.201, p-value = 0.818, F2: F_2,44_ = 1.02, p-value = 0.377). As for the expression of CI under higher temperatures, we found that a complete CI occurred even in higher temperatures (33–34 °C), and no hatched egg could be detected from incompatible crosses of infected males, and uninfected females reared in different temperatures; thus, heat has no significant effect on CI. To some degree, the number of hatched eggs in crosses of both infected males and females (w^+^ × w^+^) is higher than the hatched eggs for infected females and uninfected males (w^+^ × w^−^), which may result from the loss of the ability of infected females to restore compatibility with infected males (Fig. 2c, d).

Additionally, heat stress appears to have no significant effect on the sex ratio, which for both sets of beetles reared under optimal (30 °C) and stress temperatures (34 °C) varied between 0.5 and 0.6, confirming the suggestion that *w*Tcon has no significant role in altering the sex ratio [20, 21].

## Conclusion

Based on our results, higher temperature alters the density of *w*Tcon in the *Tribolium confusum*. We showed that among the five different temperatures beetles were reared at, the highest *Wolbachia* density was reported at 30–31 °C, which is also the most favorable temperature range for the host development, while at 34 °C, the density of *w*Tcon decreased to a great extent. Furthermore, the fertility of adult females was strongly reduced at 34 °C, with a drastic reduction in the number of laid and hatched eggs. However, based on our study (but with a rather low number of replicates), we found that CI was intact even in the mating crosses between the adult beetles, reared at the highest temperature. Therefore, this might interpret as the low-density requirement of *w*Tcon in the case of inducing CI in *T. confusum*, although this demands further experiments.

## Limitations

Changes in environmental conditions can affect the infection dynamics and the interaction of *Wolbachia* with their host and, as a result, the ability of the host to adapt in a wild population. Although our findings suggest a spatial and/or seasonal difference in *Wolbachia* densities based on our experiments, field data with wild populations would be essential to understand the effects in a natural setting. Due to time and space constraints, we limited our replicates, which should be resolved in further experiments.

## Supplementary Information

**Additional file 1.** Relative density of *w*Tcon for male and females of *T. confusum*, reared at different temperature based on Cq value for two consecutive generations as (a) F1 and (b) F2.

**Additional file 2.** Results of crosses between *Wolbachia*-infected and uninfected *Tribolium confusum*, rearing continuous at 30 °C (a), 31 °C (b), 32 °C (c), 33 °C (d), 34 °C (e). Statistical analysis by one-way ANOVA and Tukey/Kramer test (P = 0.05) (mean ± standard error).

## Data Availability

All data generated or analyzed during this study are included in this published article and its Additional files.

## Abbreviations

PCR: Polymerase chain reaction
qPCR: Quantitative polymerase chain reaction
CI: Cytoplasmic incompatibility

## References

[1] Thomas MB, Blanford S (2003). Thermal biology in insect-parasite interactions. Trends Ecol Evol.

[2] Mouton L, Henri H, Bouletreau M, Vavre F (2006). Effect of temperature on *Wolbachia* density and impact on cytoplasmic incompatibility. Parasitology.

[3] Mouton L, Henri H, Charif D, Boulétreau M, Vavre F (2007). Interaction between host genotype and environmental conditions affects bacterial density in *Wolbachia* symbiosis. Biol Let.

[4] Dunbar HE, Wilson ACC, Ferguson NR, Moran NA (2007). Aphid thermal tolerance is governed by a point mutation in bacterial symbionts. PLoS Biol.

[5] Werren JH, Zhang W, Guo LR (1995). Evolution and phylogeny of *Wolbachia*: reproductive parasites of arthropods. Proc R Soc Lond B Biol Sci.

[6] Werren JH, Baldo L, Clark ME (2008). *Wolbachia*: master manipulators of invertebrate biology. Nat Rev Microbiol.

[7] LePage DP, Bordenstein SR (2013). *Wolbachia*: can we save lives with a great pandemic?. Trends Parasitol.

[8] Gerth M, Bleidorn C (2016). Comparative genomics provides a timeframe for *Wolbachia* evolution and exposes a recent biotin synthesis operon transfer. Nat Microbiol.

[9] LePage DP, Metcalf JA, Bordenstein SR, On J, Perlmutter JI, Shropshire JD, Layton EM, Funkhouser-Jones LJ, Beckmann JF, Bordenstein SR (2017). Prophage WO genes recapitulate and enhance *Wolbachia*-induced cytoplasmic incompatibility. Nature.

[10] Saridaki A, Bourtzis K (2010). *Wolbachia*: more than just a bug in insects genitals. Curr Opin Microbiol.

[11] Bordenstein SR, Bordenstein SR (2011). Temperature affects the tripartite interactions between bacteriophage WO, *Wolbachia*, and cytoplasmic incompatibility. PLoS ONE.

[12] Perrot-Minnot MJ, Guo LR, Werren JH (1996). Single and double infections with *Wolbachia* in the parasitic wasp *Nasonia vitripennis* effects on compatibility. Genetics.

[13] Van Opijnen T, Breeuwer JA (1999). High temperatures eliminate *Wolbachia*, a cytoplasmic incompatibility inducing endosymbiont, from the two-spotted spider mite. Exp Appl Acarol.

[14] Hurst GD, Johnson AP, Schulenburg JH, Fuyama Y (2000). Male-killing *Wolbachia* in *Drosophila*: a temperature-sensitive trait with a threshold bacterial density. Genetics.

[15] Clancy DJ, Hoffmann AA (1998). Environmental effects on cytoplasmic incompatibility and bacterial load in *Wolbachia*-infected *Drosophila simulans*. Entomol Exp Appl.

[16] Hague MT, Caldwell CN, Cooper BS (2020). Pervasive effects of *Wolbachia* on host temperature preference. MBio.

[17] Wade MJ, Stevens L (1985). Microorganism mediated reproductive isolation in flour beetles (genus *Tribolium*). Science.

[18] Fialho RF, Stevens L (1996). *Wolbachia* infections in the flour beetle *Tribolium confusum*: evidence for a common incompatibility type across strains. Invert Pathol.

[19] Fialho RF, Stevens L (1997). Molecular evidence for single *Wolbachia* infections among geographic strains of the flour beetle *Tribolium confusum*. Proc R Soc Lond B Biol Sci.

[20] Kageyama D, Narita S, Imamura T, Miyanoshita A (2010). Detection and identification of *Wolbachia* endosymbionts from laboratory stocks of stored-product insect pests and their parasitoids. J Stored Prod Res.

[21] Ming QL, Shen JF, Cheng C, Liu CM, Feng ZJ (2015). *Wolbachia* infection dynamics in *Tribolium confusum* (Coleoptera: Tenebrionidae) and their effects on host mating behavior and reproduction. J Econ Entomol.

[22] Stanley MM, Grundmann AW (1965). Observations on the morphology and sexual behavior of *Tribolium confusum* (Coleoptera: Tenebrionidae). J Kansas Entomol Soc.

[23] Zhou W, Rousset F, O'Neill S (1998). Phylogeny and PCR-based classification of *Wolbachia* strains using *wsp* gene sequences. Proc R Soc Lond B Biol Sci.

[24] Zhang T, Wang YJ, Guo W, Luo D, Wu Y, Kučerová Z, Stejskal V, Opit G, Cao Y, Li FJ, Li ZH (2016). DNA barcoding, species-specific PCR, and real-time PCR techniques for the identification of six *Tribolium* pests of stored products. Sci Rep.

[25] Lee SF, White VL, Weeks AR, Hoffmann AA, Endersby NM (2012). High-throughput PCR assays to monitor *Wolbachia* infection in the dengue mosquito (*Aedes aegypti*) and *Drosophila simulans*. Appl Environ Microbiol.

[26] Lu Y, Miao S, Wang Z, Wang S (2019). Prevalence of *Wolbachia* in 10 Tenebrionidae stored-product insects and spatiotemporal infection dynamics in *Tribolium confusum* (Jaquelin Du Val) (Coleoptera: Tenebrionidae). Grain Oil Sci Tech.

