# Supplementary material for: Effect of high temperature on Wolbachia density and impact on cytoplasmic incompatibility in confused flour beetle, Tribolium confusum (Coleoptera: Tenebrionidae)
Source: BMC Res Notes. 2022 Jul 7;15:240. doi: 10.1186/s13104-022-06123-y (PMC9264519; doi:10.1186/s13104-022-06123-y)
Supplement: Supplementary file 1 — Additional file 1. Relative density of wTcon for male and females of T. confusum, reared at different temperature based on Cq value for two consecutive generations as (a) F1 and (b) F2. [file 13104_2022_6123_MOESM1_ESM.docx]

Additional file 1**.** Relative density of *w*Tcon for male and females of *T. confusum*, reared at different temperature based on Cq value for two consecutive generations as (a) F1 and (b) F2.

(a)

| F1 | Sex | x̅ Cq (Tcon) | x̅ Cq (*wsp*) | Subtract (n) | 2n |
| --- | --- | --- | --- | --- | --- |
| 30°C | **♀** | 21.68 | 18.65 | 3.03 | 8.16809701 |
| 30°C | **♀** | 22.28 | 17.5 | 4.78 | 27.474094 |
| 30°C | **♀** | 22.99 | 18.44 | 4.55 | 23.4253711 |
| 30°C | **♂** | 22.26 | 17.8 | 4.46 | 22.0086691 |
| 30°C | **♂** | 21.72 | 18.74 | 2.98 | 7.88986163 |
| 30°C | **♂** | 22.94 | 18.08 | 4.86 | 25.281322 |
| 31°C | **♀** | 23.67 | 19.53 | 4.14 | 17.6304819 |
| 31°C | **♀** | 23.61 | 18.9 | 4.71 | 26.1728659 |
| 31°C | **♀** | 23.61 | 19.2 | 4.41 | 21.258973 |
| 31°C | **♂** | 24.34 | 18.9 | 5.44 | 43.4113385 |
| 31°C | **♂** | 24.1 | 19.53 | 4.57 | 23.7523771 |
| 31°C | **♂** | 22.95 | 18.55 | 4.4 | 21.1121266 |
| 32°C | **♀** | 21.58 | 17.51 | 4.07 | 16.7954669 |
| 32°C | **♀** | 20.63 | 17.99 | 2.64 | 6.23331664 |
| 32°C | **♀** | 21.98 | 18.41 | 3.57 | 11.8761886 |
| 32°C | **♂** | 21.16 | 19.27 | 1.89 | 3.70635225 |
| 32°C | **♂** | 23.12 | 18.88 | 4.24 | 18.8958826 |
| 32°C | **♂** | 24.68 | 20.55 | 4.13 | 17.5086992 |
| 33°C | **♀** | 21.61 | 18.44 | 3.17 | 9.00046788 |
| 33°C | **♀** | 23.82 | 19.59 | 4.23 | 18.7653592 |
| 33°C | **♀** | 22.79 | 19.81 | 2.98 | 7.88986164 |
| 33°C | **♂** | 24.04 | 19.86 | 4.18 | 18.1261422 |
| 33°C | **♂** | 23.31 | 19.57 | 3.74 | 13.3614067 |
| 33°C | **♂** | 23.48 | 20.35 | 3.13 | 8.75434961 |
| 34°C | **♀** | 21.04 | 18.41 | 2.63 | 6.19025997 |
| 34°C | **♀** | 21 | 18.86 | 2.14 | 4.40762046 |
| 34°C | **♀** | 22.49 | 19.53 | 2.96 | 7.78123958 |
| 34°C | **♂** | 23.75 | 20.26 | 3.49 | 11.235559 |
| 34°C | **♂** | 22.29 | 19.32 | 2.97 | 7.83536238 |
| 34°C | **♂** | 21.81 | 18.79 | 3.02 | 8.11167584 |

(b)

| F2 | Sex | x̅ Cq (Tcon) | x̅ Cq (*wsp*) | Subtract (n) | 2n |
| --- | --- | --- | --- | --- | --- |
| 30°C | **♀** | 24.67 | 18.44 | 6.23 | 75.0614368 |
| 30°C | **♀** | 22.74 | 18.83 | 3.91 | 15.032364 |
| 30°C | **♀** | 23.22 | 18.89 | 4.33 | 20.112214 |
| 30°C | **♂** | 22.98 | 19.52 | 3.46 | 11.0043345 |
| 30°C | **♂** | 23.51 | 18.64 | 4.87 | 29.2426064 |
| 30°C | **♂** | 23.59 | 19.38 | 4.21 | 18.5070109 |
| 31°C | **♀** | 22.45 | 19.89 | 2.56 | 5.89707687 |
| 31°C | **♀** | 23.78 | 19.15 | 4.63 | 24.7610399 |
| 31°C | **♀** | 24.315 | 20.05 | 4.265 | 19.1596593 |
| 31°C | **♂** | 23.16 | 19.23 | 3.93 | 15.242208 |
| 31°C | **♂** | 23.32 | 19.53 | 3.79 | 13.8325957 |
| 31°C | **♂** | 22.72 | 20.16 | 2.56 | 5.89707687 |
| 32°C | **♀** | 22.65 | 18.84 | 3.81 | 14.0256915 |
| 32°C | **♀** | 22.06 | 18.71 | 3.35 | 10.196485 |
| 32°C | **♀** | 22.36 | 19.39 | 2.97 | 7.83536238 |
| 32°C | **♂** | 23.51 | 19.75 | 3.76 | 13.547925 |
| 32°C | **♂** | 23.39 | 19.81 | 3.58 | 11.958794 |
| 32°C | **♂** | 23.3 | 19.91 | 3.39 | 10.4831472 |
| 33°C | **♀** | 23.34 | 19.67 | 3.67 | 12.7285837 |
| 33°C | **♀** | 23.86 | 20.03 | 3.83 | 14.2214829 |
| 33°C | **♀** | 23.62 | 20.36 | 3.26 | 9.57982964 |
| 33°C | **♂** | 23.36 | 19.94 | 3.42 | 10.7034204 |
| 33°C | **♂** | 23.26 | 19.99 | 3.27 | 9.64646262 |
| 33°C | **♂** | 23.31 | 20.54 | 2.77 | 6.82107913 |
| 34°C | **♀** | 22.77 | 19.9 | 2.87 | 7.3106516 |
| 34°C | **♀** | 23.42 | 21.33 | 2.09 | 4.25748073 |
| 34°C | **♀** | 23.14 | 19.93 | 3.21 | 9.25350547 |
| 34°C | **♂** | 23.75 | 20.26 | 3.49 | 11.235559 |
| 34°C | **♂** | 22.29 | 19.32 | 2.97 | 7.83536238 |
| 34°C | **♂** | 21.81 | 18.79 | 3.02 | 8.11167584 |
