# Supplementary material for: Effect of high temperature on Wolbachia density and impact on cytoplasmic incompatibility in confused flour beetle, Tribolium confusum (Coleoptera: Tenebrionidae)
Source: BMC Res Notes. 2022 Jul 7;15:240. doi: 10.1186/s13104-022-06123-y (PMC9264519; doi:10.1186/s13104-022-06123-y)
Supplement: Supplementary file 2 — Additional file 2. Results of crosses between Wolbachia-infected and uninfected Tribolium confusum, rearing continuous at 30 °C (a), 31 °C (b), 32 °C (c), 33 °C (d), 34 °C (e). Statistical analysis by one-way ANOVA and Tukey/Kramer test (P = 0.05) (mean ± standard error). [file 13104_2022_6123_MOESM2_ESM.docx]

**Additional file 2.** Results of crosses between *Wolbachia*-infected and uninfected *Tribolium confusum*, rearing continuous at 30°C (a), 31°C (b), 32°C (c), 33°C (d), 34°C (e). Statistical analysis by one-way ANOVA and Tukey/Kramer test (P=0.05) (mean ± standard error).

**(a)**

| Temperature  (30°C) | crosses  (♂×♀) | N | number of eggs | Eggs hatched (%) | number of F1 adults | F1 females (%) |
| --- | --- | --- | --- | --- | --- | --- |
| F1 | w+ ×w+ | 3 | 60.6±3.7 | 54±15.6 | 16.5±4.8 | 58±5.5 |
|  | w- ×w+ | 3 | 68±3 | 81±5 | 28±5.3 | 49.4±10.4 |
|  | w+ ×w- | 3 | 56.6±44.4 | 0 | 0 | 0 |
|  | w - ×w- | 3 | 107.6±11 | 80±4 | 50±6.1 | 52.8±1.2 |
| F2 | w+ ×w+ | 3 | 100.6±18.4 | 90±6.5 | 46±11.3 | 54.4±3.8 |
|  | w- ×w+ | 3 | 102.3±26.57 | 82.6±14.3 | 44±19.8 | 60.1±10.8 |
|  | w+ ×w- | 3 | 76.6±10 | 0 | 0 | 0 |
|  | w - ×w- | 3 | 85.3±9.5 | 88.6±15.3 | 37±10.3 | 58.2±6.1 |

**(b)**

| Temperature  (31°C) | crosses  (♂×♀) | N | number of eggs | Eggs hatched  (%) | number of adults | Females  (%) |
| --- | --- | --- | --- | --- | --- | --- |
| F1 | w+ ×w+ | 3 | 104.6±12.5 | 82±4.5 | 59±8.8 | 56.4±2.3 |
|  | w- ×w+ | 3 | 110±13.9 | 81.6±9.29 | 55±8.8 | 53.6±3.4 |
|  | w+ ×w- | 3 | 88.6±8.6 | 0 | 0 | 0 |
|  | w - ×w- | 3 | 86.3±16 | 62±46.3 | 39±29.1 | 56.3±8.7 |
| F2 | w+ ×w+ | 3 | 105.6±32.7 | 80±8.5 | 46.3±12.8 | 46.6±2.9 |
|  | w- ×w+ | 3 | 114.6±11.5 | 87.3±4.1 | 50±7.7 | 52.8±6.5 |
|  | w+ ×w- | 3 | 92.6±36.4 | 0 | 0 | 0 |
|  | w - ×w- | 3 | 93±6.6 | 78.3±7.7 | 36.5±7 | 50.3±11 |

**(c)**

| Temperature  (32°C) | crosses  (♂×♀) | N | number of eggs | Eggs hatched (%) | number of adults | females  (%) |
| --- | --- | --- | --- | --- | --- | --- |
| F1 | w+ ×w+ | 3 | 66.6±3.2 | 81±17.3 | 27.6±6.5 | 53.4±4.3 |
|  | w- ×w+ | 3 | 89±23.2 | 79.3±6 | 38±11 | 57.2±4.2 |
|  | w+ ×w- | 3 | 69.3±3.5 | 0 | 0 | 0 |
|  | w - ×w- | 3 | 49±12.2 | 82.6±7.5 | 18±7.8 | 64.1±7.2 |
| F2 | w+ ×w+ | 3 | 69.3±3.8 | 83±6 | 28.6±4.1 | 50.8±2.7 |
|  | w- ×w+ | 3 | 97.6±12.4 | 87.6±10.6 | 42.5±9.8 | 51.7±4 |
|  | w+ ×w- | 3 | 43.3±22.8 | 0 | 0 | 0 |
|  | w - ×w- | 3 | 95.6±26.4 | 79.6±10.5 | 37.8±13.5 | 54.3±1.3 |

**(d)**

| Temperature  (33°C) | crosses  (♂×♀) | N | number of eggs | Eggs hatched (%) | number of adults | Females  (%) |
| --- | --- | --- | --- | --- | --- | --- |
| F1 | w+ ×w+ | 3 | 101.6±3 | 91.6±6.6 | 55.6±6.5 | 54.7±6 |
|  | w- ×w+ | 3 | 106.3±16 | 72± | 39.6±13.2 | 54.2±5.2 |
|  | w+ ×w- | 3 | 61.6±3.7 | 0 | 0 | 0 |
|  | w - ×w- | 3 | 94.3±14.3 | 72.6±10.7 | 38.1±16.9 | 60.5±2.3 |
| F2 | w+ ×w+ | 3 | 39.3±4 | 75±6.5 | 15.3±8.2 | 56.2±11.6 |
|  | w- ×w+ | 3 | 65±8.6 | 82.3±11.5 | 28.1±15.2 | 58.8±17.5 |
|  | w+ ×w- | 3 | 72.6±10 | 0 | 0 | 0 |
|  | w - ×w- | 3 | 86.3±27.3 | 69.3±13.1 | 33.3±19.5 | 48.8±13.4 |

**(e)**

| Temperature  (34°C) | crosses  (♂×♀) | N | number of eggs | Eggs hatched (%) | number of adults | females  (%) |
| --- | --- | --- | --- | --- | --- | --- |
| F1 | w+ ×w+ | 3 | 44.3±16.5 | 12.6±21.9 | 4.3±6.8 | 57.6±40.2 |
|  | w- ×w+ | 2 | 32.3±28.1 | 30.6±26.5 | 7.5±6 | 57.6±4.4 |
|  | w+ ×w- | 3 | 47±13 | 0 | 0 | 0 |
|  | w - ×w- | 3 | 75±3.7 | 52.3±14.2 | 20.8±6.3 | 57.3±2.4 |
| F2 | w+ ×w+ | 3 | 20.6±2.8 | 8.3±14.4 | 1±1.6 | 66.6±38.4 |
|  | w- ×w+ | 3 | 40.3±21.8 | 54±11.1 | 12.8±8.9 | 60.6±8.8 |
|  | w+ ×w- | 3 | 44±30.5 | 0 | 0 | 0 |
|  | w - ×w- | 3 | 76±12.3 | 56±8.6 | 21.1±11.4 | 57.7±10.9 |
